# Tumor Specific Epigenetic Silencing of Corticotropin Releasing Hormone -Binding Protein in Renal Cell Carcinoma: Association of Hypermethylation and Metastasis

**DOI:** 10.1371/journal.pone.0163873

**Published:** 2016-10-03

**Authors:** Hossein Tezval, Natalia Dubrowinskaja, Inga Peters, Christel Reese, Katrin Serth, Faranaz Atschekzei, Jörg Hennenlotter, Arnulf Stenzl, Markus A. Kuczyk, Jürgen Serth

**Affiliations:** 1 Department of Urology and Urological Oncology, Hannover Medical School, Hannover, Germany; 2 Department of Molecular Biology, Hannover Medical School, Hannover, Germany; 3 Department of Urology, Eberhard Karls University of Tuebingen, Tuebingen, Germany; University of Navarra, SPAIN

## Abstract

The relevance of Corticotropin Releasing Hormone (CRH)-system in human malignancies is a question of growing interest. Here we investigated hypermethylation and epigenetic silencing of the *CRH-Binding Protein (CRHBP) gene* in clear cell renal cell cancer (ccRCC). Relative methylation of the *CRHBP* CpG island (CGI) was determined in 17 tumor cell lines as well as 86 ccRCC samples and 66 paired normal tissues using pyrosequencing and quantitative methylation specific PCR of bisulfite converted DNA. Results were statistically compared with relative mRNA expression levels of *CRHBP* and clinicopathological parameters of patients. Re-expression of *CRHBP* following 5-aza-2´-deoxycytidine treatment was investigated by quantitative mRNA expression analysis. Real-time impedance analysis was applied for analysis of invasiveness of renal tumor cells following si-RNA knockdown of *CRHBP* expression or ectopic expression of CRHBP. We found the *CRHBP* CGI to be frequently methylated in tumor cell lines of renal, prostatic, and bladder cancer. Comparison of methylation in normal and paired renal cancer tissue specimens revealed hypermethylation of the *CRHBP* CGI in tumors (*p*<1*10^−12^). DNA methylation and decreased mRNA expression were correlated (R = 0.83, p<1*10^−12^). Tumor cell lines showed 5-aza-2´-deoxycytidine dependent reduction of methylation and re-expression of CRHBP was associated with altered cellular invasiveness of renal cancer cells in real-time impedance invasion assays. Hypermethylation and inverse relationship with mRNA expression were validated *in silico* using the TCGA network data. We describe for the first time tumor specific epigenetic silencing of CRHBP and statistical association with aggressive tumors thus suggesting the CRH system to contribute to the development of kidney cancer.

## Introduction

The corticotropin releasing hormone (CRH)-family includes the corticotropin releasing hormone (CRH) homologous urocortin proteins (UCN, UCN2, UCN3), their receptors CRHR1 and CRHR2 as well as the cortictropin releasing hormone binding protein CRHRB. Proteins of the CRH-system have been initially identified as hypothalamus directed mediators of neuroendocrine stress response [1, 2] while recent studies suggest that CRH family members might also play a role in the development of human solid cancers [3]. Moreover, functional studies demonstrated *in vitro* that migration of tumor cells can be enhanced by CRH protein. This stimulation in turn can be blocked by inhibition of the *ERK*-pathway [4]. Concurrent alterations in apoptotic behavior and *AKT* pathway signaling of tumor cells have also been observed following treatment by CRH and UCN2 [5]. In addition, changes in expression both of mRNA as well protein levels have been detected for CRH-family members in a number of human malignancies such as breast, endometrial, lung, prostate and kidney cancer giving further evidence for the relevance of the CRH-system in human cancers [3].

In line we recently identified changes in mRNA and protein expression levels for UCN and CRHR2 in normal and clear cell renal cell carcinoma (ccRCC) tissues [6]. A strong cytoplasmic immunopositivity for UCN was detected in normal proximal renal epithelia whereas tumor cells showed either the combination of nuclear positivity with loss of cytoplasmic signals or solely cytoplasmic immunopositivity. Furthermore, CRHR2 immunopositivity was found to be reduced in endothelia of tumoral microvessels. Interestingly, quantitation of *CRHBP* mRNA levels revealed a nearly complete loss of mRNA expression in RCC identifying a new member of the CRH-family to be possibly involved in RCC carcinogenesis [7].

The development of ccRCC is associated with loss and/or alteration of chromosome 3q and frequently observed gene mutations in the von Hippel-Lindau (VHL) and PBRM1 genes [8, 9]. Exome wide mutational analyses in substantial number of tumors on the one hand revealed a great number of additional mutations occurring in RCC [10]. On the other hand a pronounced variability of mutations was found, exhibiting nearly individual mutational spectra in tumors thus clearly limiting the clinical usability of mutational information. Interestingly, loss of VHL function was shown to associate with extended epigenetic alterations in RCC [11] and, noteworthy, the most frequent mutations described so far affect genes maintaining the cellular chromatin and histone status, a process that is interrelated with DNA methylation [12]. DNA hypermethylation in RCC has been described for a substantial number of genes, and, moreover to show functional significance in cell lines derived from RCC as well as correlation with histopathological tumor characteristics, clinicopathological parameters and course of the disease [8, 13–18].

In view of our previous findings, demonstrating that mRNA-expression of the *CRHBP* gene is depleted in tumor tissues, we hypothesized that CRHBP may be epigenetically silenced thus representing a new target of DNA hypermethylation in ccRCC. Our study shows, to our knowledge for the first time, that a member of the CRH-system can undergo epigenetic silencing in a solid human cancer, hence providing new and strong evidence for a significant role of the CRH-system in human tumorigenesis.

## Material and Methods

### Primary cells and tumor cell lines

Renal proximal tubular epithelial cells (RPTEC) and primary normal prostatic cells (PreC) were obtained from Lonza (Basel, Switzerland) and renal, urothelial and prostate cancer cell lines ACHN, A498, 786-O, RCC-GS, RCC-HS, RCC-MF, RT112, CLS439, HB-CLS2, EJ28, 5637, T24, DU145, LN-cap and PC3 were purchased from cell line services (CLS, Eppelheim, Germany). Cells were cultured according to the manufactures recommendations and exhibited a total number of 18 passages at the beginning of real-time impedance experiments.

### Patients’ characteristics

Kidney tumors with clear cell histology of 86 patients (mean age 64 years, 35–90 years) subjected to kidney surgery between 2001 and 2005 collected from the Eberhard Karls University Tuebingen and corresponding 66 tumor free tissues were included in the present study (Table 1). Tissue preparation, storage, pathological evaluation, tumor stage assessment, nuclear grading, and data management have been described previously [19]. The ethics committees "Ethik-Komission an der Medizinischen Fakultät der Eberhard-Karls-Universität und am Universitätsklinikum Tübingen (Head D. Lucht) " and "Ethik-Kommission der Medizinischen Hochschule Hannover (Head H.D. Tröger) " approved the study (ethics votes No. 128/2003V and 1213–2011) and written informed consent was obtained from patients. Organ-confined RCC was defined as pT ≤ 2 and N0/M0 and advanced disease as pT ≥ 3 and/or N+/M+.

**Table 1:** Clinical and histopathological data of patients.

Clinico-pathological parameters		Number of patients (%)All ccRCC	Number of patients (%)Paired ccRCC
		ccRCC^1^	ccRCC with pNT^2^
**Total**		86 (100)	66 (100)
**Age (years)**			
Mean		64	65
(minimum-maximum age)		(35–90)	(35–90)
	male	53 (62)	40 (61)
	female	33 (38)	26 (39)
**pT classification**			
	pT1	8 (9)	4 (6)
	pT1a	22 (26)	17 (26)
	pT1b	14 (16)	12 (18)
	pT2	6 (7)	4 (6)
	pT3	2 (2)	1 (1)
	pT3a	9 (10)	7 (11)
	pT3b/c	23 (27)	19 (29)
	pT4	0 (0)	0 (0)
	others	1 (1)	2 (3)
**Synchronous lymph nodes metastasis**			
		9 (10)	8 (12)
**Synchronous distant metastasis**			
		22 (26)	19 (28)
**Advanced disease (pT3-4 and/or N/M+)**			
		45 (52)	37 (56)
**Fuhrman grading**			
	G1	20 (23)	10 (15)
	G1-2	9 (10)	7 (10)
	G2	42 (49)	36 (54)
	G2-3	5 (6)	5 (7)
	G3	10 (12)	8 (12)

^1^ccRCC, clear cell renal cell carcinoma

^2^pNT paired normal tissue

### Nucleic acid extraction, DNA bisulfite conversion and DNA methylation analysis

Examination of control sections for tumor cell content, DNA isolation from frozen section as well as bisulfite conversion of DNA were performed as reported recently [18]. Genomic DNA from primary cells and the cancer cell lines were isolated using standard proteinase K digestion and phenol-chloroform extraction.

CRHBP CGI methylation analysis of cell lines was carried out using pyrosequencing applying the universal reverse primer concept [20]. The forward, 5´-GGAGTTGGTTGGGGAGTA -3´, reverse-universal 5´-GGGACACCGCTGATCGTTTACCCCCRCACAAAATCCCACCTT-3´ and the universal 5’-Biotin- GGGACACCGCTGATCGTTTA-3´ primers were designed using the PyroMark Assay Design 2.0 software (Qiagen, Hilden, Germany). PCR was carried out in 25 μl consisting of 60 mM Tris-HCl pH 8.5, 15 mM ammonium sulfate, 1.5 mM MgCl_2_, 0.2 mM dNTP mix, 0.5 U of HotStarTaq DNA Polymerase (Qiagen, Hilden, Germany), 50 ng bisulfite-treated genomic DNA and 0.4 μM of each primer: PCR-cycling conditions were 95°C for 15 minutes, followed by 45 cycles with denaturation at 95°C for 45 seconds, annealing at 60°C for 40 seconds and elongation at 72°C for 40 seconds finished with 1 cycle final elongation at 72°C for 5 minutes. Purification of the biotinylated PCR product, preparation of single strand DNA, annealing of the pyrocequencing primer (5´-TGGTTGGGGAGTAGT-3´) and pyrosequencing in a PyroMark Q24 system (Qiagen, Hilden,Germany) was performed according to the manufacturer´s instruction using PyroGold SQA™ Reagent Kit (Qiagen, Hilden, Germany). CpG site quantification was performed using the methylation Software PyroMark Q24.

Quantitative methylation specific PCR (qMSP) analysis of tissue specimens was carried out by use of a quantitative real-time fluorimetric 5’ exonuclease PCR assay as described previously [18]. The qMSP primers 5’- AGGGGTTGGTCGGAATCGT -3’ (forward), 5’- AACCTAAACTACGCTAAATTCCTACG -3’ (reverse) and the Taqman® probe 5’-FAM- CGCCACCCTCTCCCGCTCCTAACG -BHQ-3 were designed by use of the Beacon Designer™ software (PREMIER Biosoft, Palo Alto CA, USA).

**Fig 1:**
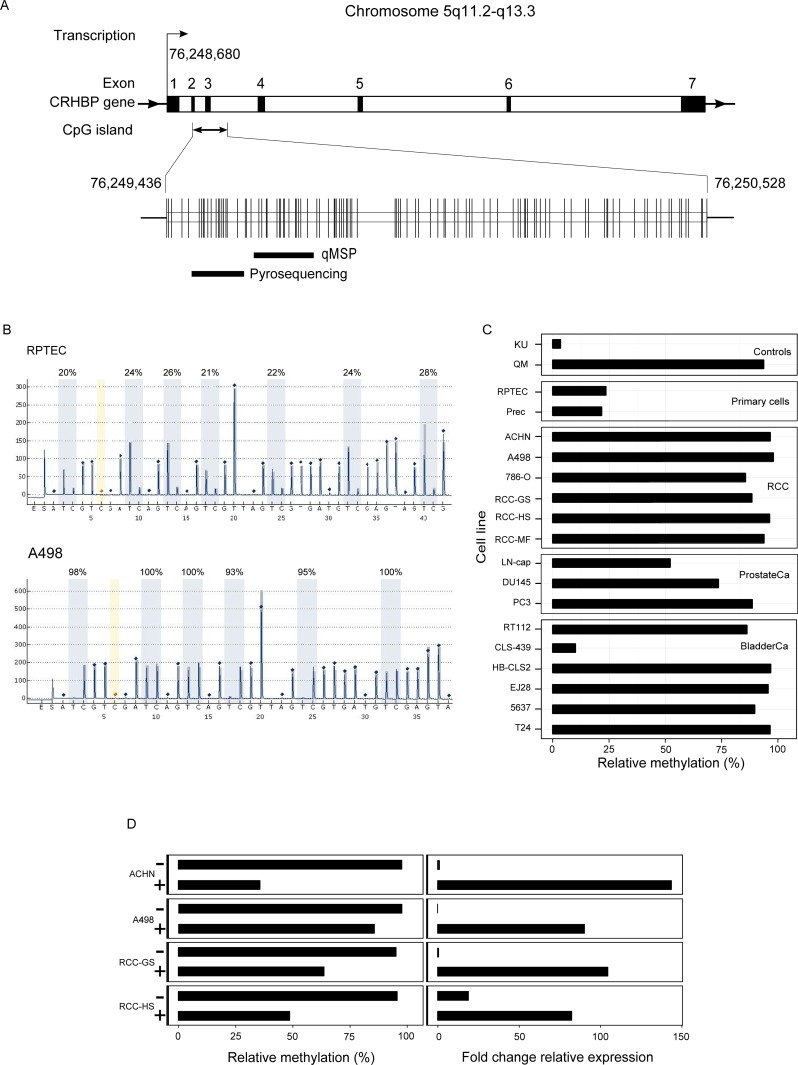
Methylation and re-expression analyses. (**A**) Structure of the CRHBP CGI and location of the PCR amplicons subjected to qMSP and pryrosequencing analysis relative to the CRHBP transcription start site. Note that only part of CpG sites covered by amplicons is amenable to quantitative methylation analysis as specified in material and methods. Vertical lines represent CpG sites within the CGI. (**B**) CHRBP pyrosequencing analysis of normal renal proximal tubular cells (RPTEC) and A498 RCC cell lines. (**C**) Pyrosequencing analysis of CRHBP methylation in controls, primary cells as well as renal cancer, prostate and bladder cancer cell lines. (**D**) Re-expression analysis of CRHBP in RCC cell lines following 5-aza-2´-deoxycytidine treatment (+/-) by the use of qMSP methylation analysis and quantitative real-time PCR of relative CRHBP mRNA expression.

We analyzed 7 and 5 CpG sites in the *CRHBP* CGI (chromosome 5: 76,249,436–76,250,528, CHRBP transcription start site at 76,248,680) at positions 76,249,507, ~510, ~512, ~519, ~526, ~536, ~541 for pyrosequencing and 76,249,622, ~628, ~683, ~696, ~720 for qMSP, respectively, as schematically illustrated in Fig 1A.

### Re-expression analysis of *CRHBP*

RCC cells were grown after thawing in normal medium (RPMI1640, 2 mM L-Glutamin, 100 μg/ml Streptomycin, 10% FCS, Biochrom, Berlin, Germany) for 7 days. After passaging cells were treated on day 2–4 with 0,125 μM 5-aza-2`desoxycytidine (Sigma, St. Louis, USA) or a mock solution (control cells) and cultured in normal medium on days 5–7 until harvesting. Isolation of total RNA, reverse transcription and relative quantitation of mRNA levels of internal controls as well *CRHBP* mRNA were carried out as described before [7].

For ectopic expression of CRHBP 3μg of human cDNA expression vector (NM_001882, Origene Technologies, Rockville, MD, USA) and PerFectin transfection reagent (T303007, Genlantis, San Diego, CA, USA) were applied according to the manufacturer´s instructions in a transfection volume of 1 ml for 6*10^5^ cells

CHO cells were used as control for transfection and expression efficiency. For SDS-PAGE and Western blot analysis each 0,5 x 10^6^ transfected cells were lysed in 100 μl 2x sample buffer (125 mM Tris-HCl pH 6,8, 20% Glycerol, 4% SDS, 5% Mercaptoethanol, 0,025% Bromphenolblue). Immunoprobing was carried out by use of the anti-Flag-POD antibody (A8592, Sigma-Aldrich, St. Louis, MO, USA).

### Real-time impedance measurement of cellular invasiveness

Considering that we detected high *CRHBP* methylation levels and correspondingly no mRNA expression in our RCC cell lines, siRNA knock down experiments for CRHBP could not be directly carried out. Therefore, the RCC-GS and–HS cell lines were first cultured using treatment with 5-aza-2´-deoxycytidine for re-expression of *CRHBP* mRNA as described above. Instead of harvesting, cells were again sub-cultured, transfected on day 2 with 25 nM Target or TargetPlus control siRNA (Dharmacon, Lafayette, USA) and use of 0.2% DH2 transfection reagent for 24 hours. Cells were then grown for 1 day in normal medium, sub-cultured, cultivated another day and counted. Each 24,000 cells per well were placed in a nutrient and growth factor deficient medium upon a 2,5% Matrigel (BD, Franklin Lakes, USA) layer in the two chamber CIM-plate system (ACEA, Biosciences, San Diego, CA, USA). Passing through the layer and a supporting membrane into the second chamber containing normal growth medium supplemented with10% FCS and growth upon the microelectrode was monitored by real-time impedance analysis by use of the XCelligence RTCA DP instrument (ACEA Bioscience, San Diego, CA, USA) and taken as a measure of cellular invasiveness. Each experiment was carried out in triplicate. Real time impedance invasion analysis of cells transfected with the plasmid for ectopic expression of CRHBP or the control plasmid was performed as described above for siRNA experiments but using each 30,000 cells per well and a Matrigel concentration of 1.5% in quadruplicate experiments.

### Statistical analysis

For comparison of kidney tumor tissues and paired tumor adjacent normal tissue samples the paired t-test was applied. Univariate logistic regression models were carried out for independent group comparisons. Means and standard deviations (sd) per group, odds ratios (OR), corresponding 95% confidence intervals (CI) and two-sided p-values are presented. Correlation of relative expression and methylation results were carried using Pearson correlation analysis. The TCGA clear cell kidney carcinoma dataset (KIRC) was applied for validation of statistical results by use of the TCGA KIRC Infinium HumanMethylation450 BeadChip and the TCGA KIRC gene expression by RNAseq (IlluminaHiSeq) level 3 data sets. Hypermethylation was statistically analyzed by use of the paired t-test. The relationship of DNA methylation and mRNA expression was evaluated using the Pearsons correlation analysis and association of methylation and clinico-pathological parameters was analyzed by the use of univariate logistic regression models. P ≤ 0.05 was considered to be statistically significant. Adjustments for multiple testing were carried out using the Bonferroni-Hochberg method.

## Results

### *CRHBP* CGI methylation analyses in cancer cell lines and primary cells

Using pyrosequencing analysis (Fig 1A and 1B) for quantitation of relative methylation levels in cells used as models for normal and tumoral human tissues, we found that RPTEC and Prec cells demonstrated methylation of approx. 20–25% (Fig 1C). In contrast, a relative methylation of 80–100% was detected for six out of six (100%) renal cancer cell lines, five out of six (83%) bladder cancer cell lines and one out of 3 (33%) prostatic carcinoma cell lines (Fig 1C). Besides CLS-439 all cancer cell lines analysed exhibited a degree of relative methylation >50% suggesting *CRHBP* CGI methylation as a frequent event in urological tumor models (Fig 1C).

### Re-expression of *CRHBP* following 5-aza-2´-deoxycytidine treatment of renal cancer cell lines

In view of our previous finding describing substantially reduced mRNA expression in renal tumors [7] we asked whether low mRNA expression of *CRHBP* in renal cancer cell lines showing a high degree of *CRHBP* CGI methylation increases due to treatment of cells with the DNA methyltransferase inhibitor 5-aza-2´-deoxycytidine. Re-expression analysis carried out for four cell lines (ACHN, A498, RCC-GS, RCC-HS) showed reduced relative methylation and a concurrent increase in relative mRNA expression levels between 50 and 150 fold following treatment of cells with the inhibitor (Fig 1D).

### Analysis of invasion characteristics of renal cancer cell lines following endogenous or ectopic re-expression of *CRHBP*

Real–time impedance analyses of cells grown on top of microelectrodes were applied to measure possible effects of CRHBP on proliferation and invasiveness of the renal cancer cell lines 786-O, RCC-GS and RCC-HS. Considering that all of these cell lines exhibited high *CRHBP* methylation and undetectable mRNA expression in QPCR analyses, we used both suppression of endogenously re-expressed CRHBP as well as targeted ectopic re-expression of CRHBP.

**Fig 2:**
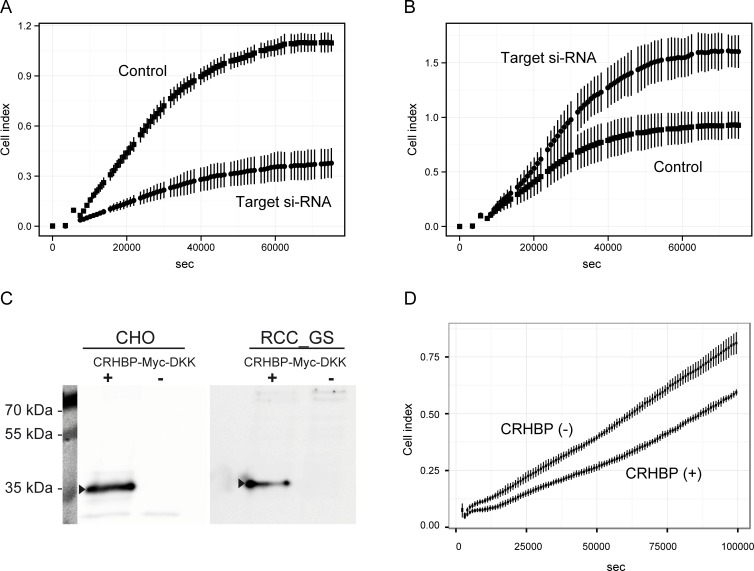
Matrigel invasion assays following CRHBP expression alterations. Real-time impedance measurement of invasiveness of the RCC-GS cell line following si-RNA-knock down of CRHBP in RCC-GS cells treated in advance with 0.125 μM (**A**) or 0.5 μM (**B**) 5-aza-2´-deoxycytidine for endogenous re-expression of CRHBP. RCC-GS cells were incubated with 25nM Target (circles) or TargetPlus control (squares) si-RNA and subsequently placed in a nutrient and growth factor deficient medium upon a membrane covered by 2,5% Matrigel layer separating the nutrient and growth factor containing second chamber. Passing through the layer and growth upon the microelectrode (cell index) was monitored against time (seconds) of measurement. Experiments were carried out in triplicate and positive and negative standard deviations of each measurement (bars) are presented. Ectopic re-expression of CRHBP following transfection of CHO control and RCC-GS target cells in western blot analysis (**C**) and the Matrigel invasion assay (**D**) using a 1.5% Matrigel layer. Single positive and negative standard deviations from quadruplicate experiments are shown for the CRHBP expression positive and negative RCC-GS cell measurements.

For demethylation and endogenous but unspecific re-expression of *CRHBP* cells were first grown in the presence of 5-aza-2`desoxycytidine and subsequently subjected to si-RNA treatment to measure the effect of CRHBP suppression upon the cellular behavior in the real-time invasion experiments. Measurements were carried out in triplicate in comparison to onTargetplus-si-RNA negative controls to monitor the invasion characteristics and indicated that CRHBP-suppression affected the invasiveness of RCC cell lines (Fig 2A and 2B). For example, the si-RNA treated RCC-GS cell line, originally derived from a metastatic primary tumor, showed a significantly increased capability to pass the matrigel-layer used as a measure for invasiveness (Fig 2A). However, changing methyltransferase inhibitor concentrations during pretreatment of cells for re-expression, we also observed reduction of the invasiveness of the cells as a result of si-RNA application (Fig 2B). Corresponding analyses for RCC-HS as a model for a localized cancer demonstrated no significant changes vs. the controls in invasion characteristics (data not shown).

Transfection of the native RCC-GS cell line with a CRHBP expression vector showed that ectopic expression of CRHBP is associated with a decreased invasive potential in the Matrigel invasion assay when compared to the RCC-GS cells transfected by use of a mock vector (Fig 2C and 2D).

### Analysis of tumor specific hypermethylation of the *CRHBP* CGI in kidney cancer specimens

**Fig 3:**
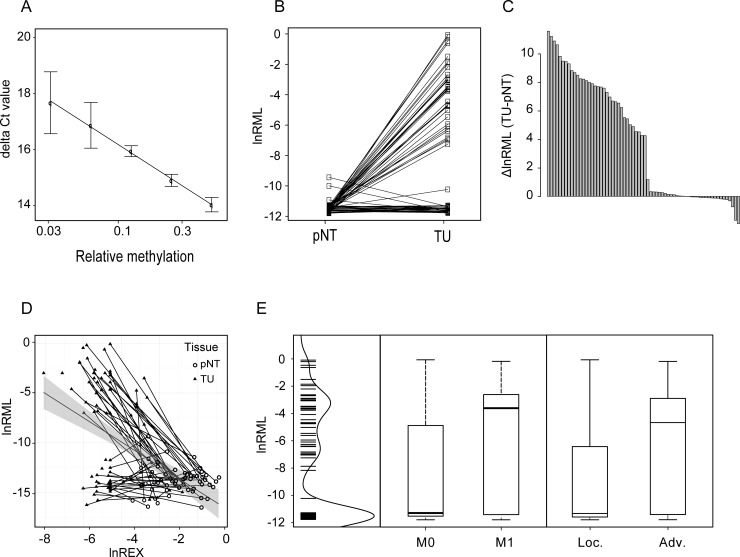
qMSP analysis of *CRHBP* CGI methylation in renal normal and tumor tissues. (**A**) Calibration line demonstrating linearity and efficiency of qMSP analysis. (**B**) Detection of hypermethylation of *CRHBP* in paired normal (pNT) and tumor samples (TU); (**C**) Assorted paired difference plot for pair-wise methylation differences; (**D**) Analysis of epigenetic silencing by comparison of relative methylation (qMSP CRHBP) as detected by qMSP and relative mRNA expression (QEXP CRHBP) as detected by quantitative real-time PCR. Paired normal (pNT) and tumor tissue (TU) samples were connected by lines. The overall regression line and 95CI (greyed area) are presented. (**E**) Analysis of association of relative methylation and metastasis and state of advanced disease. From left to right kernel density estimation of relative methylation values and boxplot presentation of metastasis negative (M0) vs. positive (M1) tumors and localized (Loc.) vs. advanced (Adv.) tumors. lnRML, natural logarithm of relative methylation, ΔlnRML, difference of natural logarithm of relative methylation for tumor (TU) and paired normal tissues (pNT), lnREX, natural logarithm of relative expression of CHRBP mRNA.

To answer whether high CGI-methylation of CRHBP associates with tumor tissues and decreased CRHBP expression in RCC, we investigated paired normal and tumoral tissue samples from 66 nephrectomy specimens by the use of qMSP. First, we characterized the qMSP assay for linearity and PCR efficiency, finding a coefficient of correlation of R = 0.99 (slope = -3.4, indicating high linearity and efficiency of the assay (Fig 3A)). Analysis of paired normal and tumor tissue samples demonstrated that more than half of the tumor tissues exhibited a substantial increase in methylation thus showing a clear overall hypermethylation of the *CRHBP*–CGI in RCC tumors (p<1*10^−12^, paired t-test, Fig 3B and 3C). Assessment of median methylation levels for normal and tumor tissues revealed a difference of 4.37 natural logarithmic units corresponding to an approx. 80 fold increase in highly methylated *CRHBP* CGI sequences in the tumor group.

### Inverse relationship of *CRHBP* CGI methylation and relative mRNA expression levels in renal cancer specimens indicates epigenetic silencing

A sample specific comparison of relative methylation detected by qMSP and relative mRNA expression values reported previously [7] showed overall a negative correlation between methylation and mRNA expression in tissue samples of ccRCC specimens (R = -0.55, p<2.7*10^−12^, Fig 3D). Both, hypermethylation data as well as the correlation analysis of methylation *vs*. expression revealed that the tumor tissues were heterogeneous with respect to CRHRBP–methylation demonstrating clearly separable subgroups of highly and low methylated tumors. A corresponding correlation analysis considering only tissue pairs with highly methylated tumors demonstrated an even higher coefficient of correlation (R = -0.83, p<1*10^−12^, Fig 3D, upper cohort).

### Association of *CRHBP* methylation and clinicopathological parameters

Statistical evaluation of methylation degree and clinicopathological parameters using univariate logistic regression revealed that higher methylation associates with both the state of distant metastasis (p = 0.01, OR 1,18) as well as advanced disease (p = 0.001, OR = 1,2; Fig 3E). Gender, age, lymph node metastasis and grade of tumors did not show a relationship with methylation of the CRHBP locus investigated (Table 2).

**Table 2:** Association of CRHBP methylation and clinicopathology of patients in RCC patients.

Parameter	*p*	OR(95%CI)
Gender	0.081	1.12 (0.99–1.24)
Age	0.490	0.96 (0.87–1.07)
Lymph node metastasis (N0 vs. N+)	0.085	1.18 (0.98–1.41)
Distant metastasis (M0 vs. M+)	0.013	1.18 (1.04–1.34)
Grade (low grade vs. high grade tumors)	0.141	1.11 (0.97–1.28)
Localized/Advanced	0.001	1.25 (1.08–1.37)

p-values and OR´s refer to statistical analyses using univariate logistic regression. Dichotomization of the parameter age was performed using the median of 64 years. Low grade (G1, G1-2 and G2) and high grade (G2-G3 and G3) definitions were applied for dichotomization of tumor grade. Localized and advanced tumor states were defined as M0 and N0 and pT<4 (localized) or M+ and/or N+ and/or pT4 (advanced).

### *In silico* validation using the TCGA KIRC data set

To independently evaluate our findings i.e. tumor specific hypermethylation, the inverse relationship between methylation and mRNA expression as well as the association of methylation with clinicopathological parameters, we interrogated the KIRC data set provided by The Cancer Genome Atlas (TCGA) research network. We found that eight out of 14 CpG sites annotated to the *CRHBP* – CGI exhibited data appropriate for statistical evaluation of paired tissue methylation. Seven of the eight loci turned out to demonstrate significant tumor specific hypermethylation (paired t-test, p-values < 1*10^−10^, Bonferroni –Hochberg adjusted for multiple testing, Table 3). Comparison of methylation with mRNA expression revealed for all of these loci a significant inverse correlation of methylation and expression (R = -0.20 to – 0.39; Table 2), Moreover, analysis of statistical associations of methylation with clinicopathological parameters showed significant relationships for five of the loci with high stage, presence of distant metastasis as well as high grade tumors (Table 3).

**Table 3:** *In silico* validation of CRHBP methylation results using TCGA KIRC data.

Locus (genomic Pos.)		^1^Hyper-methy-lation	^2^Gene silencing	^3^Clinicopathology			
				*T*	*N*	M	G
		*p*	*p*	*p*	*p*	*p*	*p*
cg06495038	(76,247,647)	6.19*10–28	2.37*10^−06^	*2*.85*10^*−05*^	-	1.04*10^−02^	1.80*10^−04^
cg26196496	(76,247,680)	3.29*10^−29^	1.03*10^−02^	*-*	-	-	-
cg01071966	(76,248,923)	3.01*10^−15^	4.98*10^−04^	*-*	-	-	-
cg04306063	(76,249,503)	9.61*10^−37^	2.32*10^−11^	*2*.34*10^*−08*^	-	7.65*10^−07^	7.45*10^−07^
cg07380705	(76,249,898)	1.34*10^−30^	2.37*10^−06^	*2*.12*10^*−06*^	-	7.56*10^−07^	1.14*10^−04^
cg13157757	(76,250,351)	5.49*10^−20^	4.42*10^−06^	*1*.69*10^*−05*^	-	2.96*10^−06^	1.11*10^−04^
cg13777717	(76,250,528)	4.21*10^−25^	2.37*10^−06^	*1*.55*10^*−06*^	-	9.49*10^−06^	5.00*10^−03^

Results are shown exclusively for cg-loci demonstrating tumor-specific hypermethylation. Specification and genomic positions of CpG sites refer to the UCSC Genome Browser on Human Feb. 2009 (GRCh37/hg19) assembly.

Only P-values considered as significant are shown for statistical associations obtained in hypermethylation, epigenetic gene silencing and clinicopathological parameter analyses

^1^paired t-test (Bonferroni-Hochberg adjusted for multiple testing) of 160 paired normal and tumoral tissues using the Methylation450k data set

^2^Pearson correlation analysis of 297 tumors (Bonferroni-Hochberg adjusted for multiple testing) using TCGA KIRC Infinium HumanMethylation450 BeadChip level 3 data and gene expression by RNAseq (IlluminaHiSeq) level 3 data sets

^3^univariate logistic regression for methylation comparison of dichotomized subsets of 284 tumors for detection of statistical association with high (> = T3) and low stage (< T3), positive or negative state of lymph node (N) and distant metastasis (M) as well as low (< G3) and high grade (> = G3) tumor subsets.

## Discussion

The potential relevance of CRH-system alterations in tumor biology has been suggested by a number of reports showing that urocortins as CRH ligands may inhibit tumor growth via effects on vascularization, promote the apoptosis of endothelial cells, and downregulate VEGF expression *in vivo* [21, 22]. In line, we recently measured a substantial reduction both of CRHBP mRNA and protein immunopositivity in ccRCC [7] thus raising the question whether epigenetic silencing of the gene could occur in tumors. So far, epigenetic alteration i.e. DNA methylation of a member of the CRH system has only been detected for the CRH gene in rats as a result of chronic stress [23]. Here we analyzed whether DNA methylation could be responsible for loss of CRHBP expression in ccRCC and associates with clinicopathological parameters of patients.

Using cancer cell lines of kidney, prostate and bladder cancers and pyrosequencing for methylation detection we found high relative methylation values in 14 out of 15 cancer cell line tumor models representing three frequent human tumors thus indicating that CGI methylation is a frequent event in human cancers. Furthermore, we found that treatment of RCC cell lines with 5-aza-2´-deoxycytidine leads to a substantial reduction of methylation and concurrent increase in mRNA expression, indicating that methylation substantially contributes in expression regulation and silencing of CRHBP in RCC cell lines. We therefore analyzed whether CRHBP shows hypermethylation in ccRCC tissue samples as well. Our quantitative methylation specific PCR analyses revealed that approximately half the tumors showed high levels of highly methylated CRHBP DNA. Corresponding to cell line analyses, a comparison of mRNA expression levels and CRHBP methylation in the tissue samples elucidated an inverse correlation of methylation and expression i.e. epigenetic silencing in renal tissues as well. Statistical evaluation of tumor methylation levels and clinicopathological parameters of patients exhibited significant associations of CRHBP methylation with the presence of distant metastasis as well as the state of advanced disease therefore indicating a role in the development of more aggressive cancer subtypes. Moreover, *in silico* validation by use of the KIRC dataset provided by TCGA network study [10] confirmed tumor specific hypermethylation of CRHBP, epigenetic silencing of CRHBP mRNA expression and association of CRHBP methylation with biological aggressive tumor characteristics.

Our analysis of the proliferation and invasion characteristics of RCC cancer cell lines following both si-RNA knock down of endogenously re-expressed CRHBP after 5-aza-2´-deoxycytidine treatment as well as ectopic re-expression of CRHBP in an epigenetically silenced RCC cell line interestingly revealed substantial alteration in the matri-gel invasion assay. In case of siRNA knock down the extent and direction of measured effects depended on the concentration of the methylation inhibitor. However, considering that treatment of cells with 5-aza-2´-deoxycytidine changed expression of an unknown number of other genes and therefore new derived cells with altered microscopic and assumingly also molecular characteristics developed, a specific reaction to CHRBP level changes could be theoretically explainable. On the other hand, ectopic expression of CHRBP also showed alteration, i.e. reduction of cellular invasiveness thus providing further evidence that invasive behavior of renal cancer cells may be affected by CRHBP. Nonetheless, all of our functional analysis are still preliminary and require future functional validation using improved tumor models taking into account the complexity of the CRH signaling network.

It has been reported previously that CRH promotes migration of squamous epithelial tumor cells [4]. In prostatic cells it has been found that CRH and Ucn2 affect apoptosis of tumor cells [5]. Moreover it has been demonstrated that the CRH ligand Ucn promotes hepatic cancer cell migration by up-regulating cPLA2 expression via CRHR1 whereas it suppressed tumor cell migration by down-regulating iPLA2 expression via CRHR2 [24]. While these studies identify members of the CRH system to participate in skin and prostatic tumor model cell lines, our preliminary functional analyses provide evidence that the CRH system may also be of relevance for kidney tumors cells.

Conclusively, our analysis clearly demonstrated epigenetic silencing of CRHBP hence showing for the first time that a member of the CRH-system is epigenetically silenced in tumor cells and thus providing strong evidence for an involvement of CRH members in human tumorigenesis. Moreover, we found association of DNA methylation with aggressive ccRCC tumor subsets, a finding that appears in nearly perfect concordance with the *in silico* results provided by the TCGA network. Consequently, both analyses statistically point to CRHBP silencing as a factor contributing to the development of aggressive tumors. Our introductory functional analysis suggests that CRHBP may affect invasive behavior of kidney tumor cells therefore possibly explaining the statistical results. Beside implications for oncological research our results may be also relevant for physiological conditions considering that most genes identified to be epigenetically silenced in tumors already show e.g. age dependent methylation in normal tissues, possibly affecting gene function [23, 25].

To expand our pathophysiological understanding of CRHBP function in tumor biology and ccRCC as well, future studies will rely on identification of improved cell line models to resolve the interplay of CRH peptides, receptors, binding protein and epigenetic alteration in human tissues.
